# Stress distribution patterns during the gait cycle in patients with anterior femoral notching following total knee replacement

**DOI:** 10.1186/s12891-022-05643-9

**Published:** 2022-07-28

**Authors:** Jin-Cheng Zhang, Le-Shu Zhang, Hang Zhou, Wang Chen, Zheng-Hao Hu, Xiang-Yang Chen, Shuo Feng

**Affiliations:** Department of Orthopedic Surgery, Affiliated Hospital of Xuzhou Medical University, 99 Huaihai Road, 221002 Xuzhou, Jiangsu China

**Keywords:** Total knee replacement, Finite element analysis, Anterior femoral notching, Gait cycle, Biomechanics

## Abstract

**Background:**

Anterior femoral notching (**AFN**) is a severe complication of total knee replacement (**TKR**), which in a percentage of patients may lead to fractures after surgery. The purpose of this study was to investigate the stress distribution in patients with AFN and the safety depth of AFN during the gait cycle.

**Methods:**

We performed a finite element (**FE**) analysis to analyse the mechanics around the femur during the gait cycle in patients with AFN. An adult volunteer was selected as the basis of the model. The TKR models were established in the 3D reconstruction software to simulate the AFN model during the TKR process, and the 1 mm, 2 mm, 3 mm, 4 mm, and 5 mm AFN models were established, after which the prosthesis was assembled. Three key points of the gait cycle (0°, 22°, and 48°) were selected for the analysis.

**Results:**

The stress on each osteotomy surface was stable in the 0° phase. In the 22° phase, the maximum equivalent stress at 3 mm was observed. In the 48° phase, with the increase in notch depth, each osteotomy surface showed an overall increasing trend, the stress range was more extended, and the stress was more concentrated. Moreover, the maximum equivalent force value (158.3 MPa) exceeded the yield strength (115.1 MPa) of the femur when the depth of the notch was ≥ 3 mm.

**Conclusions:**

During the gait cycle, if there is an anterior femoral cortical notch ≥ 3 mm, the stress will be significantly increased, especially at 22° and 48°. The maximum equivalent stress exceeded the femoral yield strength and may increase the risk of periprosthetic fractures.

## Background

Anterior femoral notching (AFN) during total knee replacement (TKR) has been considered to be a potential high-risk factor for periprosthetic fractures (PPFs) [1, 2]. Due to the fact that AFN weakens the cortex of the femur, it will cause sudden changes in the stress of the anterior femur, which can easily result in postoperative knee kinematics disorders, as well as shortening the life of the prosthesis, and easily causing PPFs [3]. In recent years, a clinical study has shown that patients with AFN were 17 times more likely to develop PPFs than patients without AFN [3, 4].

The incidence of AFN in TKR has been reported to be approximately 1%. However, incorrect surgical procedure may significantly increase this incidence to a staggering 30–40% [2, 5, 6]. In recent years, the application of modern equipment has reduced the incidence of AFN [7]. Even though the incidence of AFN has decreased, it is still a nonnegligible problem in surgery. AFN can occur due to various factors such as the incorrect selection of prosthesis size, posterior displacement of the osteotomy module and abnormal femoral anatomy [7–9].

Previous studies [3, 10] have shown that AFN causes a peri-femoral stress increase, especially when the AFN is ≥ 3 mm. Based on the FE analysis, the stress distribution around the femur during the gait cycle when AFN occurs has never been previously studied. Therefore, the purposes of this study were to investigate the stress distribution in patients with AFN and to examine the safe depth of the AFN during the different gait cycles.

## Methods

### Geometry

An adult volunteer participated in the experiment and signed an informed consent form (a female who was 60 years old; height: 165 cm; weight: 67.8 kg; body mass index: 24.9 kg/m^2^; cortical bone thickness: approximately 4 mm). In this study, the knee joint of a healthy adult was selected for 3D reconstruction. The knee joint model was optimized and subsequently received a simulated osteotomy of the knee joint to create a post-TKR model of a normal knee joint. For the osteotomy method, the distal osteotomy was a 6-degree external rotation osteotomy with 10 cm of bone being intercepted; the femur was rotated via external rotation to align with the surgical epicondylar axis (3°), the sagittal position was parallel to the mechanical axis of the distal femur, and an anterior cut, posterior cut, and anterior and posterior chamfer cuts were performed via a four-in-one osteotomy module. Vanguard PS series implants (Zimmer Biomet, Warsaw, USA) were used for all of the models. Model building and simulation of the surgery were performed by using a combination of MIMICS (Materialise Interactive Medical Image Control system, Belgium; Version 21.0) and SOLIDWORKS (Dassault Systèmes, USA; Version 2018) software. This model was validated by using a previous model [10] for the comparative validation of the condylar stress distribution.

**Fig. 1:**
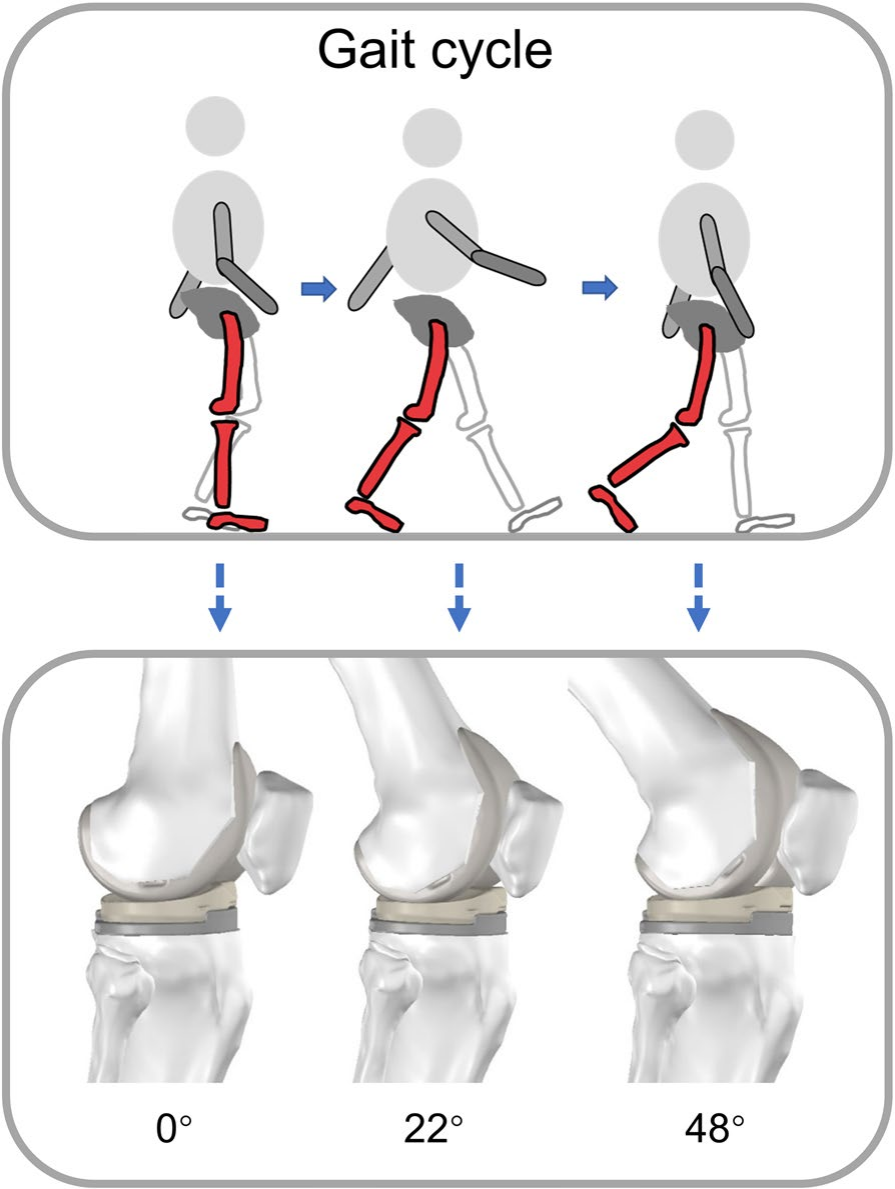
Schematic diagram of the gait cycle and corresponding knee joint model

Based on the normal knee joint model, we translated the osteotomy surface, into a 1 mm interval according to previous studies [10]. Five groups of anterior cortical notch models of 1 mm, 2 mm, 3 mm, 4 mm, and 5 mm depths were established, as shown in Fig. 1. Additionally, the normal TKR knee models were included. In this study, a series of six model groups were enrolled.

### Material properties and conditions

It was assumed that the femoral prosthesis, the femoral stem, the cement layer and the cancellous bone region exhibited linear elasticity, isotropy and uniformity. All of the interfaces were defined as fully bound, with the implant fully tied to the outer cement surface and the cement layer fully tied to the femoral surface [11, 12]. Based on previous studies and population values [13–15], the weight was standardized, and the material values were assigned. In this study, the values of Young’s modulus and Poisson’s ratio that were applied to all of the structures are shown in Table 1.

**Table 1:** Assignment of material properties applied to the finite model

Component	Young’s modulus E (N/mm2)	Poisson’s ratio (*ν*)
Cortical bone	16,700	0.3
Cancellous bone	600	0.3
Cement	2280	0.3
Femoral component (Co–Cr)	210,000	0.3

### Loading and boundary

**Fig. 2:**
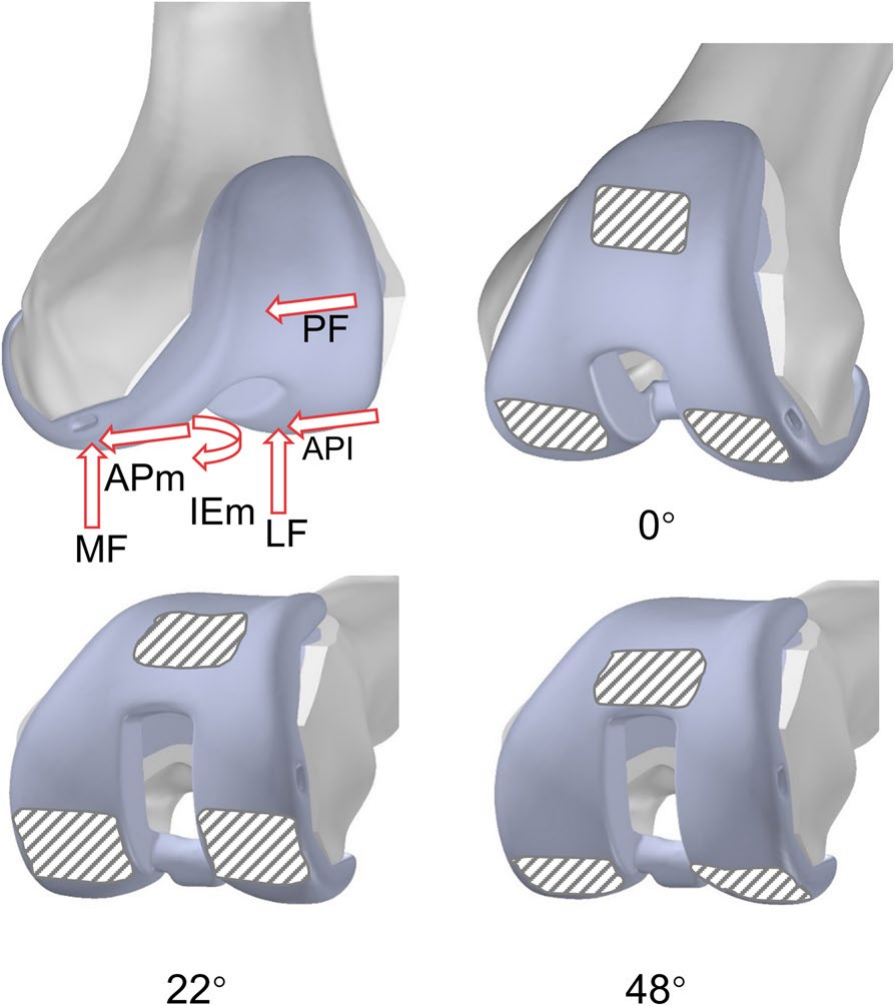
Schematic diagram of the direction and location of force on the knee joint (the red arrow shows the direction of the force; the grey slash area is the area of the contact surface)

Due to the fact that the reported loading of the knee joint for the same exercise has been shown to vary greatly [16–18], this investigation considered the forces operating on the knee as reported by prior studies using in vivo telemetric implants [19, 20]. To enable the generation of consistent datasets, load values were normalized in terms of the subject’s body weight. Three phases of the normal gait cycle (0°, 22°, and 48° of knee flexion) were included in this study (Fig. 1). 0° referred to the vertical stance phase of the gait cycle, 22° was the intermediate phase of knee flexion of the gait cycle, and 48° was the maximum load phase of the gait cycle [11, 21]. The load acting on the femur consisted of six separate parts [22]: the patella-femoral force (PF), the normal force of the medial and lateral joints (MF/LF), the anterior-posterior force of the medial and lateral joints (APm/APl), and the internal/external moment (IEm) [14]. The values of each force for the three phases are detailed in Table 2. The determination of the contact surfaces consisted of the following two aspects: the contact surfaces reported in previous studies [11, 12], and the display of the overlap surface function in the software to determine the contact surfaces of the medial and lateral femoral condyles as well as the patella at different angles. By combining the two observations, the location of the contact surfaces was confirmed. All of the forces were applied as distributed pressure loads to the contact area at each angle (Fig. 2).

**Table 2:** Forces used in the FE analyses for the three flexion angles

Force	0°	22°	48°
Patella-femoral force (PF)/N	45	327	567
Normal force of the medial (MF)/N	436	1159	1160
Normal force of the lateral (LF)/N	291	772	773
Anterior-posterior force of the medial (APm) /N	-57	130	-3
Anterior-posterior force of the lateral (APl) /N	-57	130	-3
Internal/external moment (IEm)/Nmm	-829	3292	-7029

To ensure data accuracy, the maximum allowable element edge length for all of the models was 2 mm. The FE meshes consisted of quadratic tetrahedron elements (C3D10 M). The simulation run time for each model was approximately half an hour. Moreover, all of the simulation calculations were based on a computer with a six-core AMD Ryzen processor and 16 GB of RAM.

### Measurement index

**Fig. 3:**
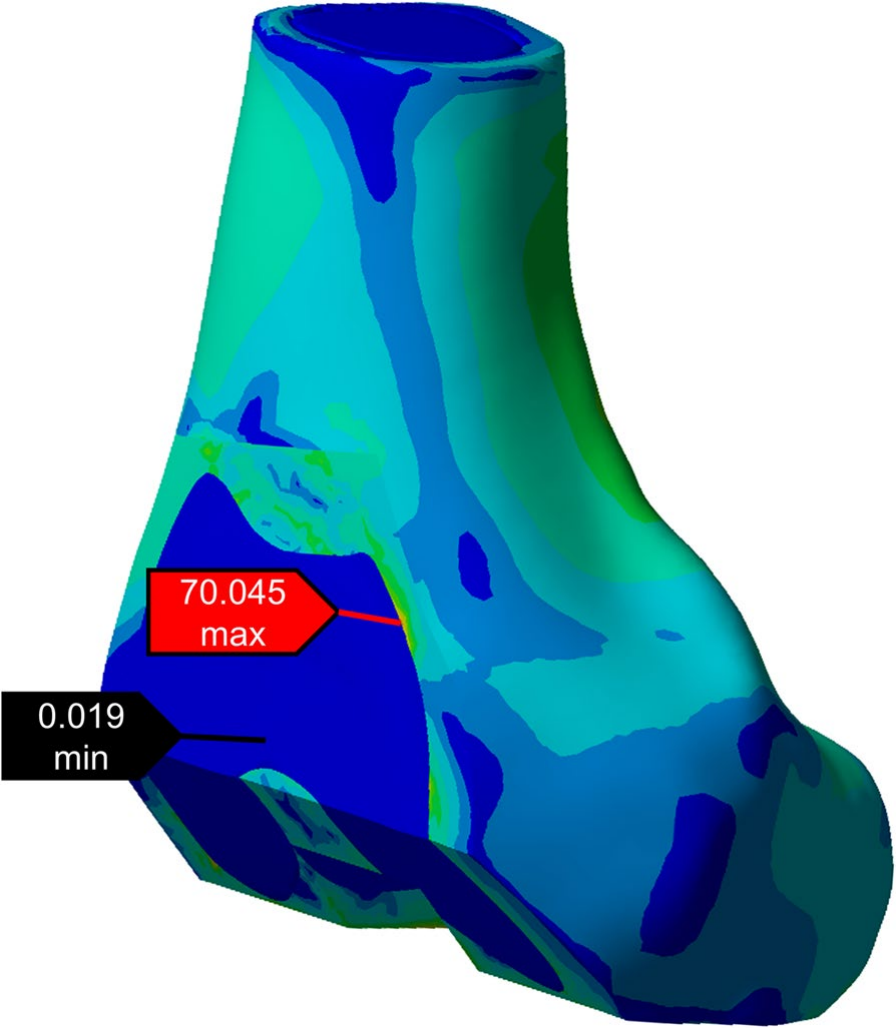
Measurement of the equivalent (von-Mises) stress in the software (the red label is the maximum equivalent stress value of the anterior condyle osteotomy surface, and the black label is the minimum equivalent force value of the anterior condyle osteotomy surface)

To compare the maximum equivalent (von Mises) stress during the gait cycle for each group of osteotomy surfaces (Fig. 3), we included the anterior condylar osteotomy surface (AC) (anterior condylar surface and notch surface), the anterior chamfer surface (AS), the distal osteotomy surface (D), the posterior chamfer surface (PS), and the posterior condylar osteotomy surface (PC).

## Results

The model was validated against the previous model [10], and the anterior condyle stress distribution and stress values were found to be similar.

**Fig. 4:**
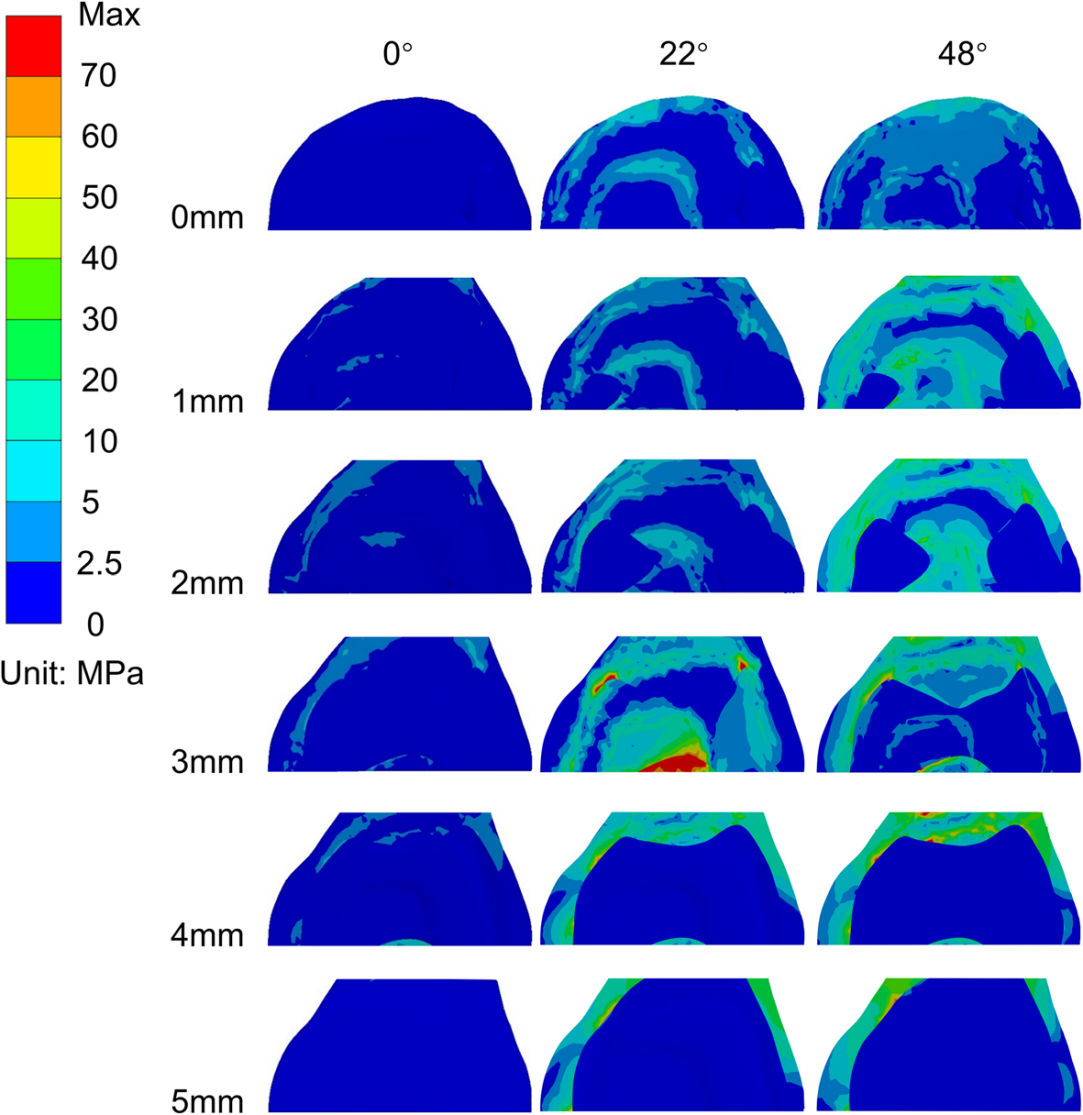
The maximum equivalent (von-Mises) stress diagram of the anterior condylar osteotomy surface at various angles

**Fig. 5:**
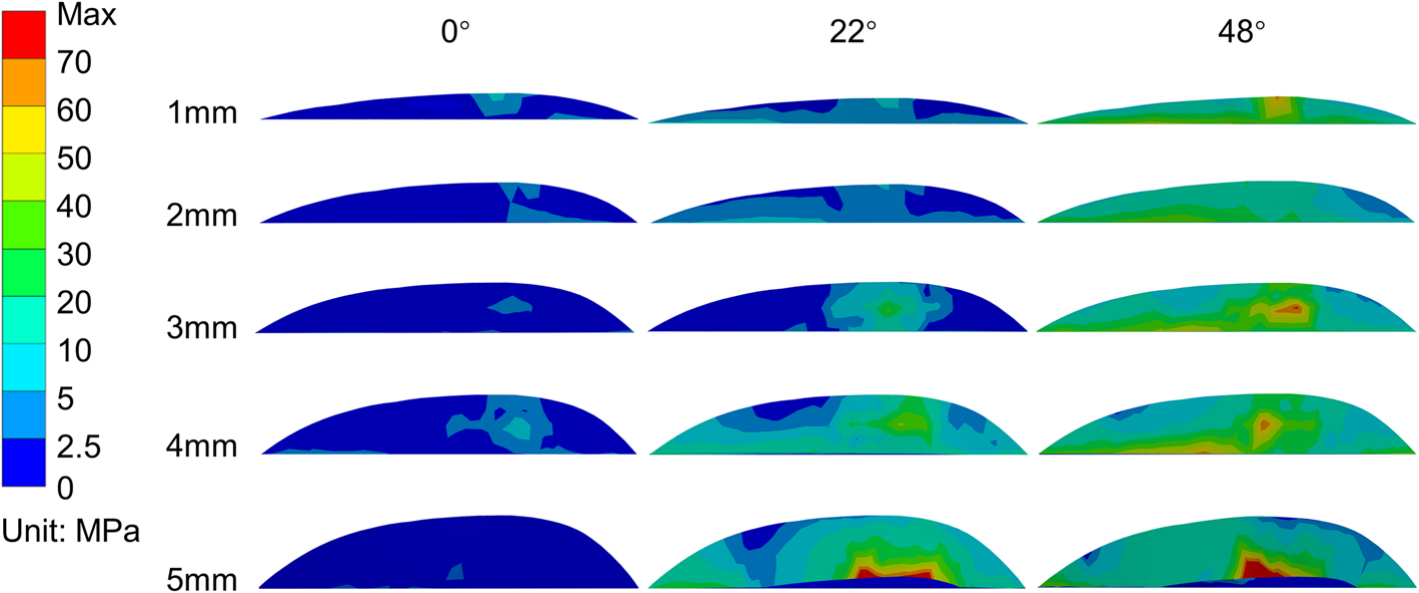
The maximum equivalent (von-Mises) stress diagram of the anterior condylar osteotomy surface (notch surface) at different angles

**Fig. 6:**
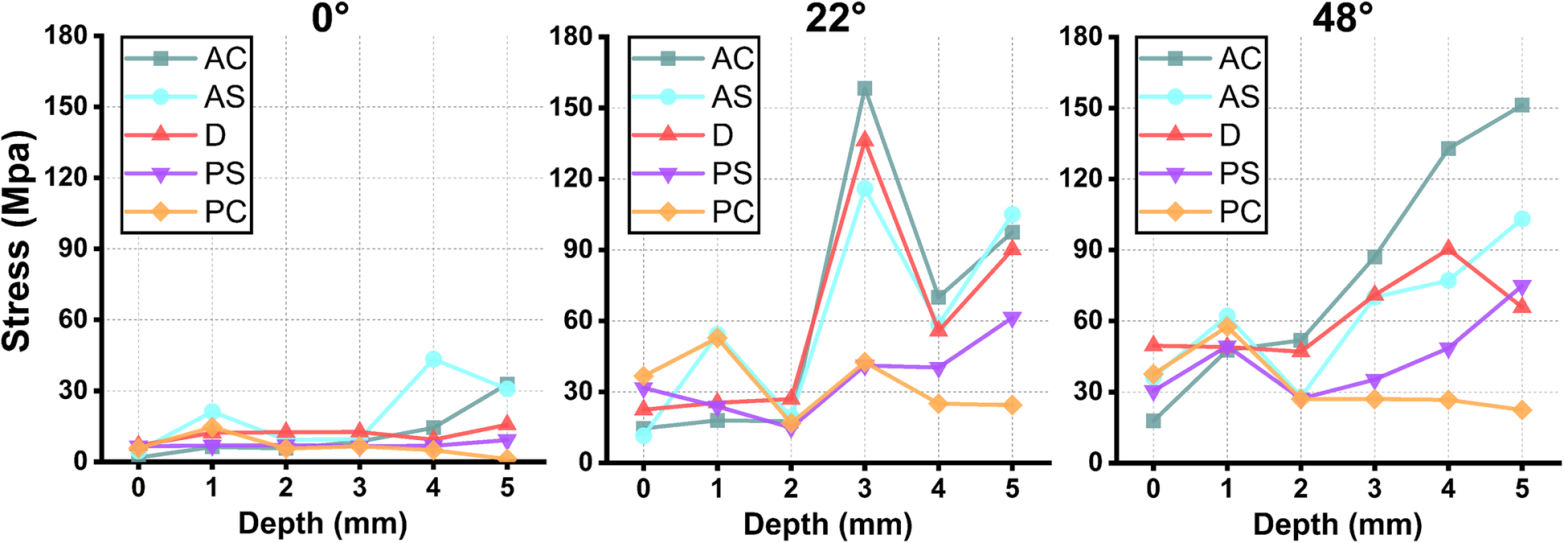
Equivalent stress of each osteotomy surface of the knee joint at different angles (AC, anterior condylar osteotomy surface; AS, anterior chamfer osteotomy surface; D, distal osteotomy surface; PS, posterior chamfer osteotomy surface; PC, posterior condylar osteotomy surface)

As shown in Figs. 4 and 5, and Fig. 6, in the 0° phase, the stress at AC was small and tended to increase with increasing notch depth. For the three osteotomy surfaces D, PC, and PS, the stresses exhibited little tendency to change with increasing notch depth and were stable in the fixed interval. For AS, the stresses at 4 and 5 mm exhibited larger stresses.

In the 22° phase, the stresses exhibited an overall increasing trend at D, AS, and AC as the depth of the notching increased and showed the maximum stress at 3 mm (158.3 MPa). Compared to the nonnotching model, the stress increased by 1087.1%. Moreover, the maximum equivalent stress exceeded the yield strength of the femur (115.1 MPa) [23, 24]. For PS and PC, the stress distribution was stable.

In the 48° phase, when the depth of cortical notching was shallow at AC, there was already a concentration of stress; as the depth increased, the stress was greater and more extensive (the range shifted from the anterior condylar surface to the notch surface), as shown in Fig. 5. By the time it reached 5 mm, the extent of the stress had reached the area where the prosthesis was in contact with the cortex in the notch surface, and the maximum stress was 151.2 MPa, which increased by 851.4% compared to the nonnotching model, thus exceeding the yield strength of the femur [23, 25, 26]. For D, the stresses did not vary considerably up to 3 mm and significantly increased beyond 3 mm. For PS, the stress variation fluctuated at 0–2 mm, whereas the stress increased subsequently after more than 2 mm. For PC, the stress decreased and became irregular. All of the equivalent stress data are shown in Fig. 6.

## Discussion

The most significant finding of this study was that the anterior condylar osteotomy surface, the distal osteotomy surface and the anterior chamfer surface were subjected to extremely large and extensive stress concentrations in patients with an anterior femoral cortical notch of 3 mm or greater, especially at 22° and 48° of knee flexion, which exceeded the yield strength of the femur (115.1 MPa). Our results have implications for clinical practice: AFN should be avoided as much as possible during TKR. Patients with AFN ≥ 3 mm should be given more attention in postoperative radiograph follow-ups and rehabilitation exercises.

Our results showed that in the 0° phase, the stresses on each osteotomy surface tended to increase, but none of them exceeded 50 MPa. In the knee without notching, the stress was also within the range of 50 MPa. Previous studies [24] on the material mechanics of bone have shown that the tensile yield stress of the femur was 71.6 MPa and the compressive yield stress was 115.1 MPa. Consequently, our study showed that the maximum stress did not exceed the femoral yield stress when the AFN was between 0 and 5 mm during the stance phase, and the stress on the femur was tolerated in both the tensile and compressive directions for the knee joint.

**Fig. 7:**
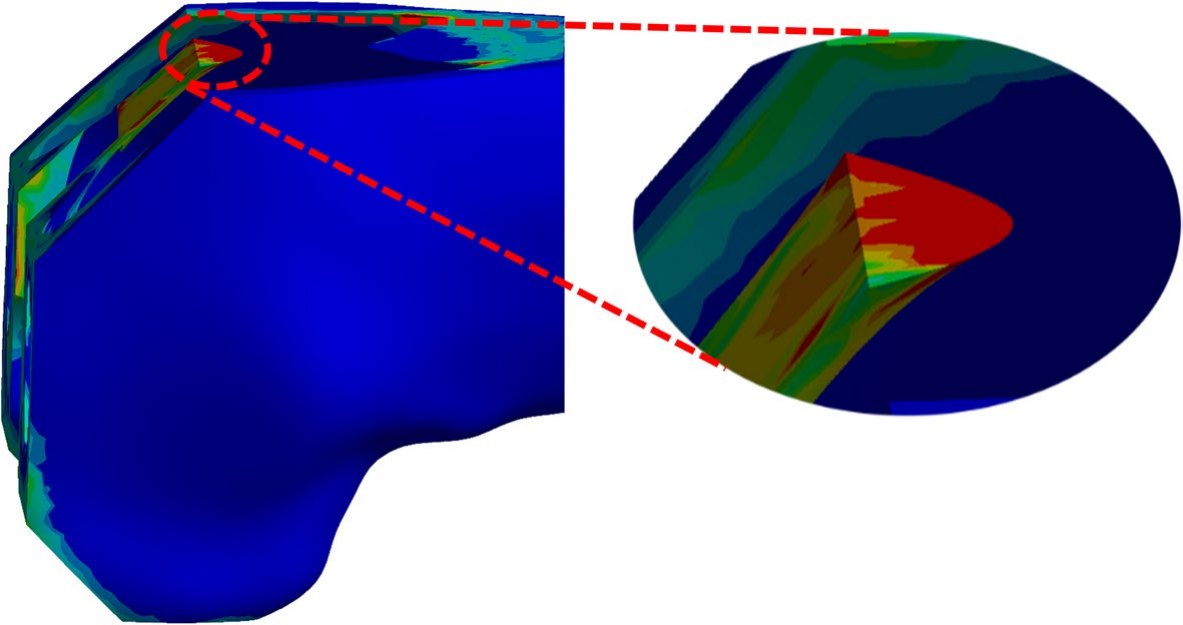
Enlarged schematic view of the cortical cancellous bone junction

In this study, the maximum stresses in the 22° and 48° phases were 158.3 MPa, and 151.2 MPa, respectively, which increased by 1087.1% and 856.3%, respectively. We observed that the stress concentration points in our model almost appeared at the junction of cortical cancellous bone (Fig. 7), and the stress concentration points gradually shifted to the notch surface as the depth of the notching increased. In the deeper 4 and 5 mm models, the osteotomy surface reached directly to the cancellous bone layer, wherein the stress zone expanded and the stress was slightly decreased. In a study by Zalzal et al. [10], it was similarly found that notching occurred at the transition from cortical bone to cancellous bone, thus generating the maximum stress. This result was in agreement with our conclusions.

Studies [24, 27] of the mechanical properties of bone have shown that the compressive yield strength of the femur was 115.1 MPa, beyond which there was a degradation of the material properties; specifically, microdamage of the bone occurred, which may indicate an important role of microdamage in increasing the risk of fracture. In addition, in the studies by Completo [12] and Martin [28], they showed that the strain increase effect at the notch edge (combined with an osteopenia bone environment) were likely to lead to an injury process of fatigue fracture. In our model, the maximum stress of the anterior femoral condyle was 158.3 MPa, which exceeded the compressive yield strength of the femur. Therefore, in clinical settings, AFN may lead to microdamage of the femur during the gait cycle, especially in the 22° and 48° phases. Even though it was lower than the compressive limit stress (205 MPa) [24], the microdamage of the bone that occurred may still increase the risk of fracture in patients with AFN.

In clinical practice, AFN is often considered to be a high-risk factor for PPFs [3, 6, 8, 10, 29, 30]. The majority of reported PPFs in patients with AFN are due to falls [3, 30]. However, in a clinical study, Lee et al. [30] reported that a patient developed AFN during TKR and gradually showed signs of PPFs on follow-up radiographs. In addition, Kinney et al. [31] reported that a patient with AFN developed PPFs with no history of trauma. In this study, the stress exceeded the femoral yield strength in patients with AFN during the gait cycle, potentially leading to fatigue fracture and progressive signs of PPFs. Therefore, combined with our experimental results, we suggest that for the clinical guidance of postoperative rehabilitation of patients with AFN, more attention needs to be paid to the radiograph follow-ups compared to patients following TKR so that possible fractures can be detected and corrected in a timely manner, thus reducing the time and cost for patients.

Previous biomechanical studies [10, 12, 32] have shown that the anterior femoral cortical area was exposed to a large stress concentration when AFN ≥ 3 mm. A recent meta-analysis and systematic review study on AFN [3] showed that patients with an anterior femoral cortical notch ≥ 3 mm had higher risks of PPFs complications. Combined with the results of the present study, these results showed that patients with anterior femoral cortical notch ≥ 3 mm have stresses that exceed the yield strength of the femur, thus increasing the possibility of PPFs.

Model validation and sensitivity analysis are very important for finite element models [33]. Model validation ensures the accuracy of the model results through direct and indirect validation and determines the impact of model condition values on target results through a sensitivity analysis. Previous studies [34, 35] have shown that a sensitivity analysis of finite element models requires consideration of multiple factors, including material properties such as bone, ligament, and prostheses, implant-prosthesis alignment, the degree of internal and external rotation of the knee joint, and the number of meshes. The forces that were implemented in this study were derived from forces measured in in vitro experiments. The effects of the tibia, medial and lateral collateral ligaments, and anterior and posterior cruciate ligaments were reduced in the model. Moreover, prosthesis alignment was performed by using standard TKA procedures, and mesh quantities were also normalized (2 mm), which reduces possible numerical errors due to model conditions.

### Limitations

First, this experimental study was conducted by using a healthy female, and although the material assignment and model selection referred to previous population values and are more similar to the real human model, there may still be limitations in its application to all individuals. Second, the simulation model does not incorporate the effects of muscles and ligaments. Third, the model was simplified to be linearly elastic, isotropic, and homogeneous, and these assumptions were made to understand and assess the stress distribution of the knee joint in patients with AFN. In addition, there is a mismatch between patient-specific geometry and a standardized, nonpatient-specific loading scenario. A sensitivity analysis was not performed in this study. Therefore, our results need to be interpreted with caution.

## Conclusions

During the gait cycle, if there is an anterior femoral cortical notch ≥ 3 mm, the stress will be significantly increased, especially at 22° and 48°. The maximum equivalent stress exceeded the femoral yield strength and may increase the risk of periprosthetic fractures.

## Data Availability

All data generated or analyzed during this study are included in this published article.

## Abbreviations

AFN: anterior femoral notching
TKR: total knee replacement
FE: finite element
PPFs: periprosthetic fractures
PF: the patella-femoral force
MF: the normal force of the medial
LF: the normal force of the lateral joints
APm: the anterior-posterior force of the medial joints
APl: the anterior-posterior force of the lateral joints
IEm: the internal/external moment
AC: anterior condylar osteotomy surface
AS: anterior chamfer surface
D: distal osteotomy surface
PS: posterior chamfer surface
PC: posterior condylar osteotomy surface
